# The evolution of SPHIRE-crYOLO particle picking and its application in automated cryo-EM processing workflows

**DOI:** 10.1038/s42003-020-0790-y

**Published:** 2020-02-11

**Authors:** Thorsten Wagner, Stefan Raunser

**Affiliations:** Department of Structural Biochemistry, Max Planck Institute of Molecular Physiology, Otto-Hahn-Strasse 11, 44227 Dortmund, Germany

**Keywords:** Cryoelectron microscopy, Software

## Abstract

Particle selection is a crucial step when processing electron cryo microscopy data. Several automated particle picking procedures were developed in the past but most struggle with non-ideal data sets. In our recent Communications Biology article, we presented crYOLO, a deep learning based particle picking program. It enables fast, automated particle picking at human levels of accuracy with low effort. A general model allows the use of crYOLO for selecting particles in previously unseen data sets without further training. Here we describe how crYOLO has evolved since its initial release. We have introduced filament picking, a new denoising technique, and a new graphical user interface. Moreover, we outline its usage in automated processing pipelines, which is an important advancement on the horizon of the field.

## Abstract (web summary)

Wagner and Raunser recently presented a deep learning based particle picking program for Cryo-EM, crYOLO. Here they discuss recent improvements to the program, a graphical user interface and share their thoughts on desired future developments.

## The crYOLO particle picking procedure

A major goal of electron cryo microscopy (cryo-EM) is to obtain high-resolution three-dimensional (3D) reconstructions of proteins and protein complexes to gain novel biological insights. This process involves the selection of thousands to millions of noisy two-dimensional (2D) particle projections, a number that only keeps increasing with recent advances in hardware and software development.

In our recent work in *Communications Biology*^1^ we introduced the “crYOLO” particle picking procedure. It is based on a deep neural network and the You Only Look Once (YOLO) object detection framework^2^. This approach enables the automated picking of particles within cryo-EM micrographs with a low signal-to-noise ratio requiring minimal human supervision or intervention. CrYOLO is easy to configure and train on a specific data set. It is fast and can process up to six micrographs per second. As crYOLO sees the complete micrograph, it is able to learn the overall context of the particles. Therefore, the approach enables highly accurate picking, e.g., it does not select particles on the carbon film or specifically picks particles attached to liposomes. In addition, a pretrained, generalized model further allows the selection of particles in previously unseen data sets with high accuracy.

## Recent evolution of crYOLO

**Fig. 1:**
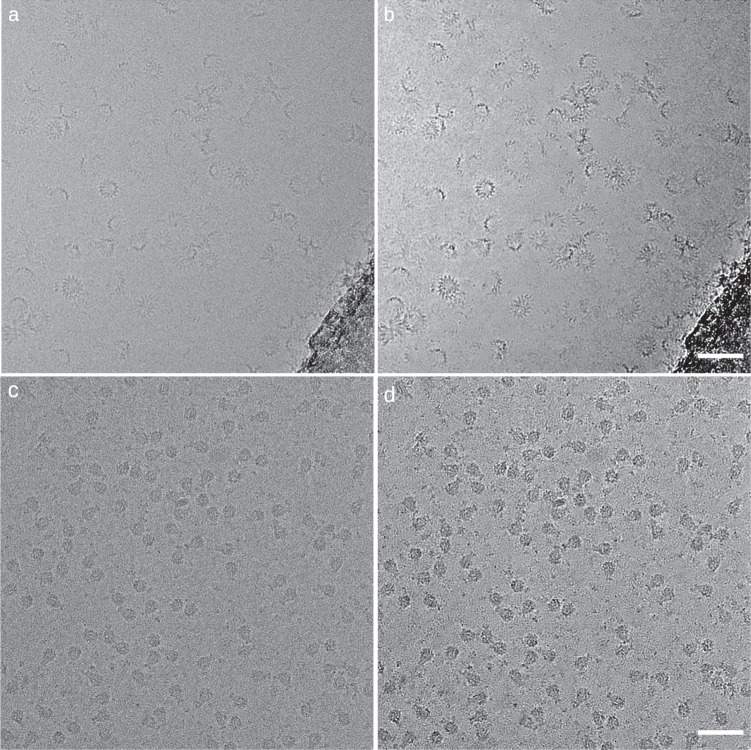
Example micrographs denoised by JANNI. XaxAB toxin^32^ and Tc toxin^33^ without denoising (**a**, **c**) and with denoising (**b**, **d**), respectively. Details for the sample and grid preparation can be found in ref. ^32^ for the XaxAB toxin and in ref. ^33^ for the Tc toxin. Scale bars: 50 nm.

Since the release of crYOLO we have improved the software by modifying the network architecture, adding new functionalities, and increasing its usability. In particular, we have integrated a new method for denoising micrographs to increase the signal-to-noise ratio for improved particle detection. By default, crYOLO uses a standard low-pass filter for denoising. However, this method requires parameters to be manually set and has its inherent limits. To enable automated denoising, we therefore implemented the recently introduced neural-network-based approach noise2noise^3^ into a new tool called JANNI, that can be chosen in crYOLO as alternative denoising method. We pretrained JANNI on movies from various cryo-EM data sets and used it to denoise previously unseen data sets (Fig. 1). JANNI might be helpful especially for data sets with low signal-to-noise ratio.

**Fig. 2:**
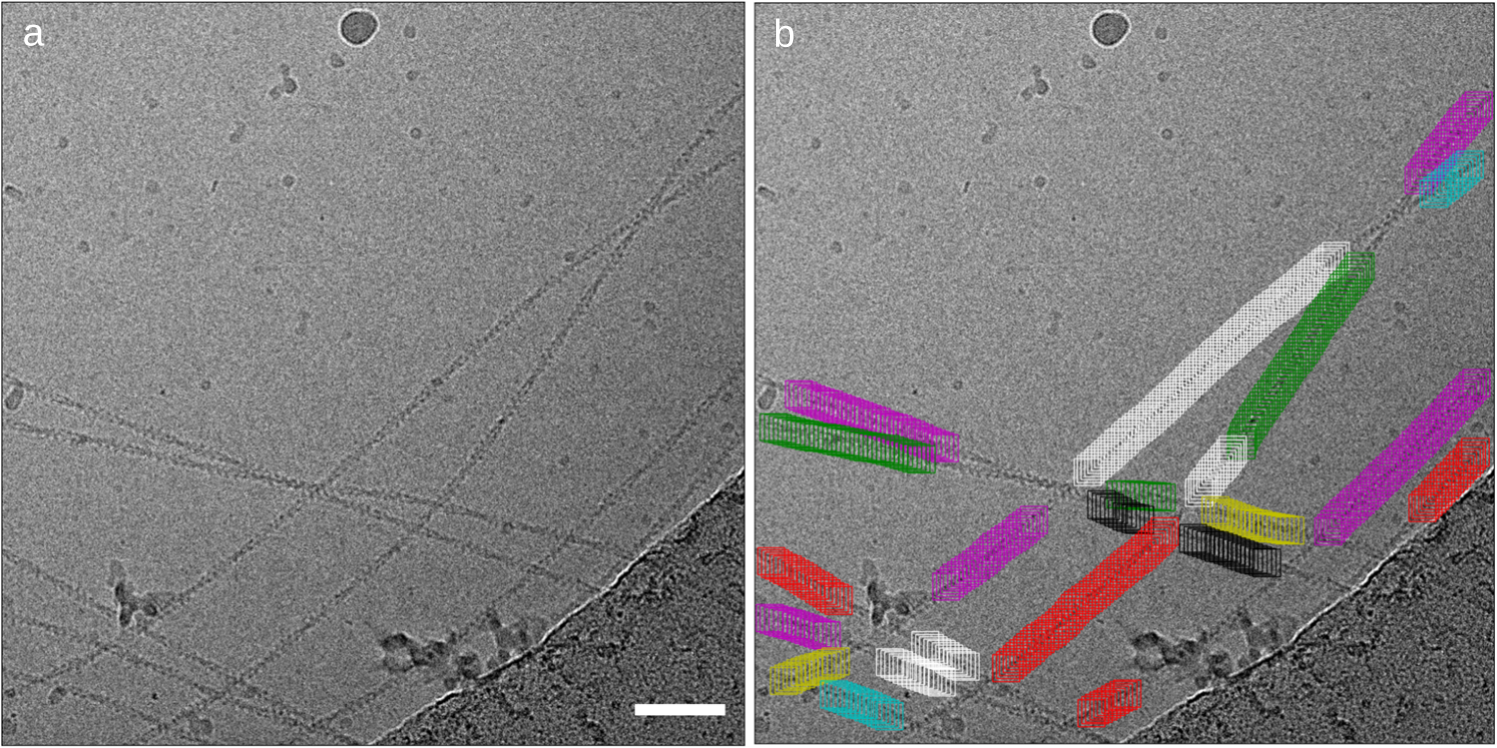
Actin picked with crYOLO filament mode. **a** Sample micrograph with actin filaments. **b** CrYOLO learned to skip crossings of filaments, contaminations and the carbon edge. Details for the sample and grid preparation can be found in ref. ^34^. Scale bar: 50 nm.

Another important new functionality of crYOLO is filament picking. Owing to their structure, the picking of filaments poses a challenge and is often not supported by automated particle picking procedures. Optimally, only single filaments are selected and positions where filaments cross or overlap are omitted. In case of helical specimens, the boxes should be placed along the filament in a distance according to its helical rise to allow the use of helical reconstruction procedures^4^. The new filament picking procedure initially follows the general workflow of crYOLO. In a post-processing step, it uses the picked particles as support points to trace the filaments. The boxes are then placed along the filaments in a distance defined by the user (Fig. 2).

CrYOLO offers now the possibility to improve an existing model, which is of advantage when fine-tuning a general model on a specific data set. In this case, only the last few layers of the network are retrained while previous layers are fixed. This effectively reduces the amount of training data needed to improve a working model. A major advantage of this approach is a substantial speed-up along with reduced GPU memory consumption.

**Fig. 3:**
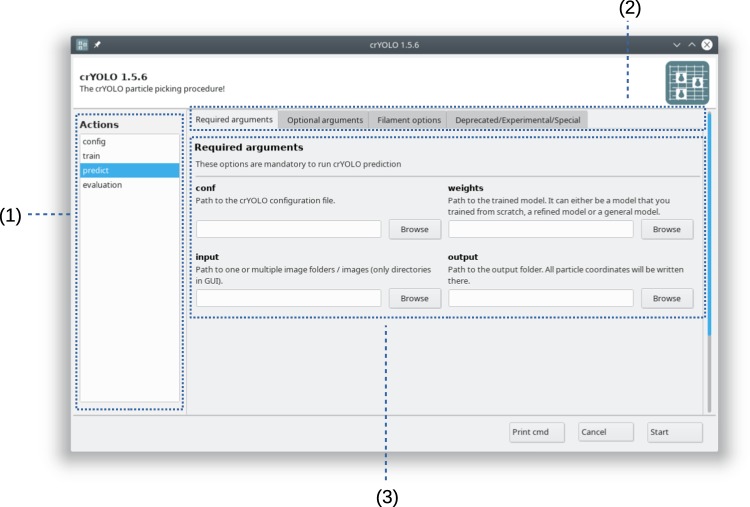
Graphical user interface (GUI) of crYOLO. The GUI is divided into three parts: (1) List of all available actions like training a model or the prediction of particles on micrographs. (2) Groups of options for the respective action. (3) Available options for the selected action.

With the evolution of crYOLO, more options have become available to the user, which increases the complexity of the command line interface. Therefore, we most recently added a new graphical user interface, which makes crYOLO more accessible for new or less technically oriented users (Fig. 3).

## Impact of crYOLO

CrYOLO has found widespread use, and since its release mid-2018 already >15 structures were solved with the support of crYOLO^5–22^. For example, Pang et al.^23^ used crYOLO to selectively pick particles, which were attached to liposomes; Rogala et al.^14^ highlighted in their study about mTORC1 that crYOLO was especially useful to exclude particles on carbon; Joppe et al.^24^ made use of crYOLO in a streamlined pipeline for rapid structure determination of yeast fatty acid synthase; and the new filament mode was recently used by Pospich et al. to examine the structural effects of toxins on actin filaments^16^.

In addition, crYOLO was made available through the SBGrid software collection^25^, enabling easy access to crYOLO for groups without advanced computational facilities. CrYOLO was also integrated into COSMIC^26^, a web platform for cryo-EM data processing via cloud computing. Very recently, Li et al.^27^ used crYOLO in a user-free preprocessing pipeline. This shows that crYOLO has been broadly used by other groups and proven flexible enough for a wide variety of applications.

## The general model and automated processing

Since CrYOLO provides a generalized model, it is the optimal particle selection software to be integrated in an automated cryo-EM single-particle analysis procedure. The general model of crYOLO was pretrained on >60 different data sets, including proteins of various sizes and shapes. This allows to pick previously unseen particles not included in the training data set. To this end, crYOLO is a crucial part in our software package SPHIRE that we are optimizing to be used in a completely automated fashion^28^. In Scipion, crYOLO is supported for the construction of intelligent workflows^29^. A recent integration of crYOLO into the automatic pipeline of Relion^30^ is successfully used at the Electron Bio-Imaging Center (eBIC) at Diamond Light Source^31^.

Whereas the generalized model offers great opportunities for automated processing there remain limitations. The amount of data used for the general model is still limited and might be biased towards the set of proteins used for the initial training. The general model is also not able to distinguish between intact and dissociated or fragmented particles in the same sample. This requires additional training to fine-tune the general model with particles manually picked from a few micrographs. A drawback is that this requires manual intervention and is therefore not suitable for automated processing. A better strategy is to automatically fine-tune the general model based on 2D classification, where particles representing similar views are grouped together, aligned and averaged.

During 2D classification, broken particles will be separated from intact particles. The latter ones will then be used to train a crYOLO model or fine-tune the general model.

Optimally, a fully automated pipeline would also include a deep-learning-based 2D class selection tool. Our group is currently developing such software, that we call Cinderella. While it is still under development, it is already publicly available and successfully integrated in SPHIRE^28^. Cinderella provides a pretrained general model and is able to separate 2D classes into good and bad classes.

In the future, a combination of Cinderella and crYOLO will allow automated feedback loops to improve the picking quality in an iterative manner. With these tools at hand, we believe that real-time automated processing even for challenging data sets is in reach.

## Data Availability

All data supporting the findings of this study are available from the corresponding author on reasonable request.
